# Disseminated Kaposi sarcoma presenting in unusual locations: A case report

**DOI:** 10.4102/sajr.v21i2.1260

**Published:** 2017-11-14

**Authors:** Adziambei Mudau, Nonjabulo Z. Makhanya, Farhana E. Suleman

**Affiliations:** 1 Department of Radiology, University of Pretoria, South Africa

## Abstract

Kaposi sarcoma (KS) is the most common malignancy associated with HIV infection. It usually affects the skin, the gastrointestinal tract and the lungs. It is generally described in the setting of CD4 counts < 150 cells/mm^3^ – 200 cells/mm^3^. We describe a case of recurrence of KS with a rare presentation of breast and musculoskeletal involvement in the setting of a CD4 count of 374 cell/mm^3^ and an undetectable viral load. The patient was on highly active antiretroviral therapy for 5 years at the time of the second presentation.

## Introduction

Kaposi sarcoma (KS) is a vascular lesion of low-grade malignant potential associated with human herpes virus 8 (HHV-8).^1^ It is the most common malignancy associated with HIV infection.^2^ It usually affects the skin, the gastrointestinal tract and the lungs.^1^

There have also been reports of KS in unusual locations; breast, gonads, adrenal glands, thyroid glands, bones and skeletal muscles.^1^ We report a case of an HIV-positive patient with disseminated KS involving the breast, skeletal muscle and bone.

## Case report

A 42-year-old HIV-positive woman with a CD4 count of 374 cell/mm^3^ and an HIV viral load lower than detectable, presented to mammography with masses in the left breast and lower back. She had a past medical history of KS with skin lesions on the face and in the lower limb, treated with chemotherapy 5 years previously. No skin lesions were noted at this presentation. She was on highly active antiretroviral therapy (HAART) since her previous presentation.

**FIGURE 1:**
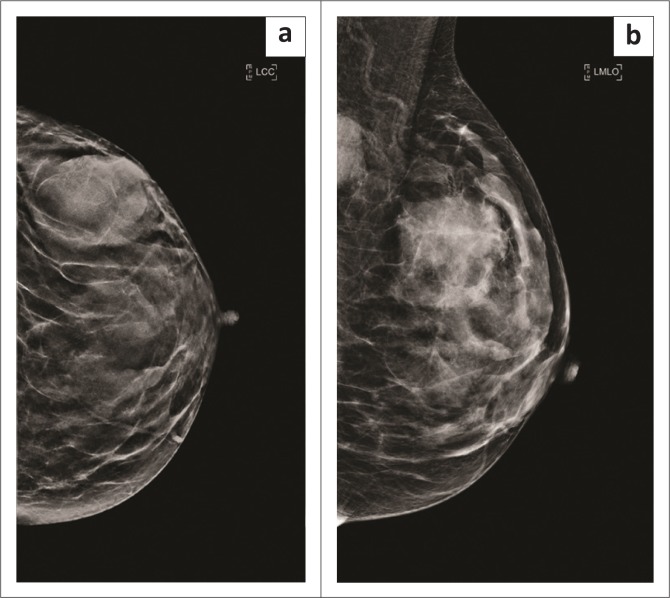
(a) Craniocaudal and (b) mediolateral oblique mammogram views of the left breast demonstrating a round mass lesion in the upper outer quadrant and a pathological node in the axilla.

**FIGURE 2:**
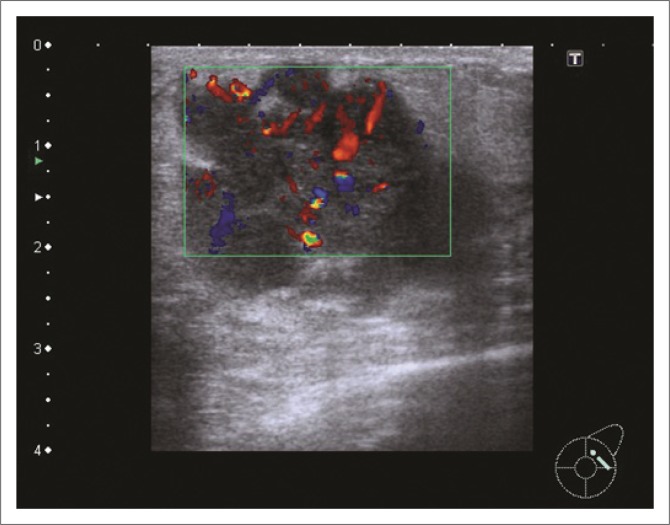
Ultrasound image of the hypoechoic mass in the left breast demonstrating posterior enhancement and increased vascularity.

Mammography revealed a large round mass in the superolateral quadrant of the left breast (Figure 1). It had obscured margins, and no calcifications were noted within the mass. Ultrasound showed a heterogeneous mass with increased vascularity and discrete margins measuring 36 mm × 24 mm (Figure 2). There were pathological intramammary and axillary lymph nodes present. An ultrasound-guided core biopsy of the breast mass was performed using a 14-gauge needle. The lower back mass was also biopsied.

Immunohistological findings of the masses were consistent with KS. HHV stain was strongly and diffusely positive.

**FIGURE 3:**
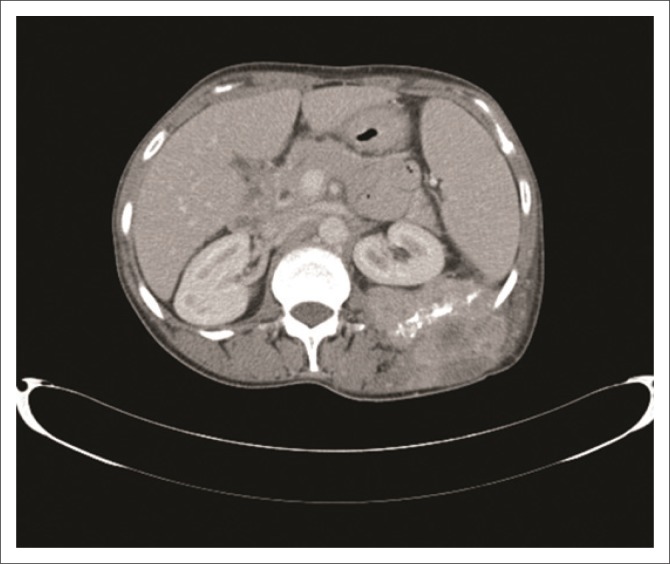
Axial post contrast computed tomography scan image at the level of the 12th rib demonstrating the destruction of the left rib with surrounding soft tissue mass.

The patient then had a computed tomography (CT) scan from the base of the skull to the symphysis pubis, which showed a heterogeneously enhancing left breast mass, a left lower back mass infiltrating the iliocostalis and quadratus lumborum muscles with encasement and destruction of the 11th and 12th ribs (Figure 3) as well as a lytic lesion in the 11th thoracic vertebra. Further lesions were noted in the liver and spleen with widespread lymphadenopathy.

## Discussion

In 2015, the World Health Organization (WHO) reported that 36.7 million people were living with HIV.^3^ Sub-Saharan Africa remains the most severely affected, with nearly one in every 25 adults (4.4%) living with HIV and accounting for nearly 70% of the people living with HIV worldwide.^3^ The estimated overall HIV prevalence rate is approximately 12.7% of the total South African population. The total number of people living with HIV in South Africa is estimated at approximately 7.03 million in 2016.^4^

The risk for KS in acquired immunodeficiency syndrome (AIDS) patients is estimated to be 20 000 times greater than the general population and 300 times greater than in other immunocompromised populations.^5^

Kaposi sarcoma is a low-grade vascular tumour that typically manifests as one of the four variants: classic KS, endemic KS (Africa), iatrogenic KS (organ transplant-related) and AIDS-related KS.^2^ KS is the most common malignancy associated with HIV infection, and it is an AIDS defining malignancy.^5^ It is associated with HHV-8, and the virus infects the KS spindle cells.^1,5^ AIDS-related KS develops in the setting of a low CD4 lymphocyte count < 150 cells/mm^3^–200 cells/mm^3^.^2^ However, there is a single article in the literature that mentions that it can manifest at any time in the course of HIV infection.^5^

Frequently KS manifests in mucocutaneous sites, typically the skin of the lower extremities, face, trunk, genitalia and oropharyngeal mucosa.^1^ The lymph nodes and visceral organs, most commonly respiratory and gastrointestinal tracts, can also be involved.^1^ The manifestations of KS have changed considerably with the advent of HAART. As a result of HAART, a smaller proportion KS patients present with visceral disease.^5^

In cases of localised disease, treatment with HAART alone could result in improvement or resolution of disease. This is thought to be directly attributable to the suppression of HIV replication and a decrease in viral load with an increase in CD4 lymphocyte count.^6^ Disseminated disease, however, would require a combination of HAART as well as chemotherapy.^6^

An immune reconstitution inflammatory syndrome (IRIS) has also rarely been reported in patients who are HAART naïve at the time of KS diagnosis. This may vary in severity, and fatal cases have been reported.^6,7^ This was unlikely to be the cause of recurrence in our patient as she had been on HAART for 5 years before her second presentation.

Kaposi sarcoma of the breast is rare. In our literature search, we found three reported cases of KS in the breast, two with disseminated disease^5,8^ and one with isolated breast involvement.^5^ Only one of the cases reported had concurrent bone involvement^1^ and one case occurred in the setting of low CD4 counts < 100 cells/mm^3,5^ Our case presented with the involvement of two unusual sites: breast and musculoskeletal system in the setting of good immunity with a CD4 count of 374 cells/mm^3^ and an undetectable viral load. The patient was also on HAART at the time of this presentation.

## Conclusion

The HIV pandemic has resulted in an increased incidence of KS occurring in common sites described above. Our patient presented with multiple rare findings that included involvement of the breast and musculoskeletal system in the setting of good immunity. There are minimal reports in the literature of such cases.
